# Knowledge of Eating Disorders and Relative Energy Deficiency in Sports among Public Health Nurses at Elite Sport Schools

**DOI:** 10.1177/23779608251407784

**Published:** 2026-01-13

**Authors:** Siri Heradstveit, Lisa Garnweider-Holme, Brita Askeland Winje, Jorunn Sundgot-Borgen, Therese Fostervold Mathisen

**Affiliations:** 1 Faculty of Health Sciences, 60499 Department of Nursing and Health Promotion, Oslo Metropolitan University, Oslo, Oslo, Norway; 2 Norwegian School of Sports Sciences, 25567 Institute of Sports Medicine, Oslo, Oslo, Norway; 3 Faculty of Health, Welfare and Organisation, 558033 Østfold University College, Fredrikstad, Norway

**Keywords:** Public health nurse, adolescent athlete, eating disorders, physical health, mental health

## Abstract

**Introduction:** Eating disorders (EDs) are increasingly common, and their prevalence among athletes is approximately three times higher than in the general population. In athletes, EDs can be more difficult to detect, yet they carry serious clinical consequences, particularly due to their association with Relative Energy Deficiency in Sport (REDs), characterized by impaired health and performance. **Objective:** This study investigated public health nurses’ (PHNs) knowledge and perceived competence when working with athletes at elite sport middle and high schools in identifying and managing EDs and REDs. The effect of years with work experience and course attendance was investigated, and we also explored their preferred course format. **Methods:** This cross-sectional study invited PHNs from all elite sport middle and high schools in Norway to respond to a questionnaire in 2025. A total of 58 PHNs were invited, of which 22 (37.9%) consented to participate and responded to a digital survey. **Results:** The findings indicated that PHNs have limited knowledge of REDs and its underlying cause, low energy availability. In contrast, their understanding of EDs was generally satisfactory. Notably, prior attendance in subject-specific courses was associated with significantly greater knowledge of REDs (g = 1.1, *p* *=* *0.03*), while years of professional experience showed no such effect. PHNs expressed a preference for “meet-the-expert” courses over self-directed learning materials such as books and reports. **Conclusion:** These results highlight the need for targeted education, and interdisciplinary collaboration between schools and PHNs, to equip PHNs with the specialized competence and position required to support adolescent athletes effectively.

## Introduction

The World Health Organization has placed eating disorders (EDs) among the priority mental health disorders among children and adolescents (World Health Organization, 2024). In recent years, the prevalence of EDs among adolescents has reportedly increased worldwide (Dahlgren et al., 2023a; Galmiche et al., 2019; Reas et al., 2025; Silén & Keski-Rahkonen, 2022; Surén et al., 2022). Of particular concern, research from the past three decades indicates that elite athletes (aged ≥16 years) have a prevalence of disordered eating (DE, i.e., the condition is not severe enough to meet the full criteria for a clinical diagnosis) and EDs that is two to three times higher than the general population (Bratland-Sanda & Sundgot-Borgen, 2013; Herpertz-Dahlmann, 2015; Keski-Rahkonen & Mustelin, 2016a).

Highlighted causes to this increased prevalence of EDs among athletes are manifold. Athletes often face high expectations regarding their body appearance (Mathisen et al., 2023; Mountjoy et al., 2023). Also, in many sports, body mass plays a critical role in performance, such as in gravity sports, where excess weight must be carried over distances or overcome with power, or in esthetic sports, where appearance significantly impacts evaluation (Mountjoy et al., 2023; Sundgot-Borgen et al., 2025).

Concurrently, a problematic culture has developed in sports where frequent measurements of body composition have become normalized. Worrying, this is associated with an increased focus on body appearance and physique, and consequently, a higher risk of DE and EDs (Mathisen et al., 2023). Other sport-specific factors that trigger EDs may be environmental or contextual ones, like a new coach, new team, or increased competitive level. These factors may lead athletes to pursue performance enhancements and conform to athletic ideals by attempting to alter their body mass or composition, often through dieting (Ghazzawi et al., 2024; Mathisen et al., 2023).

Adolescent athletes are particularly vulnerable, as performance demands increase during the transition from children's sports to adolescent sports. This is specifically underlined by findings of more frequent symptoms of EDs in *adolescent* athletes than non-athletes of both genders across all sports and competition levels (Mancine et al., 2020). And recently, a study found a consistently elevated rate of EDs in young elite and recreational athletes over a 12-year period (2009–2021) (Martinsen & Sundgot-Borgen, 2013; Sundgot-Borgen et al., 2025).

## Review of Literature

DE and EDs increase the risk of health issues, as they co-occur with or induce mental and physical health impairments (Keski-Rahkonen & Mustelin, 2016b). In sports medicine, the term low energy availability (LEA) describes a condition where the energy intake is too low to cover the basic energy requirement when exercise takes too much of the energy supply (Mountjoy et al., 2023). Adolescent athletes are specifically vulnerable to such issues, as they need increased energy to support growth, development in addition to the exercise strains (Reece et al., 2023), and the exercise energy expenditure typically increases as they progress to elite sport middle and high schools. Acknowledging the high prevalence of DE and EDs among young athletes, alongside the relevant risk factors, young athletes are at specific risk for LEA (Mountjoy et al., 2023). This may ultimately place young athletes at high risk for the sport-specific syndrome called relative energy deficiency in sports (REDs). According to the International Olympic Committee (IOC) consensus statement from 2023, REDs is a clinically diagnosed, multifactorial syndrome characterized by a collection of negative health and performance outcomes resulting from exposure to problematic LEA (Mountjoy et al., 2023).

Young athletes find themselves in a split between trusting the guidance and advice they receive from parents, school, and sports. Professional expertise on health challenges is expected to be found among health personnel, such as public health nurses (PHNs) associated with the school health service. PHNs are a part of the Norwegian school support system around adolescent athletes and have a professional role in promoting optimal health and identifying adolescents with symptoms of health issues (The Norwegian Directorate of Health, 2017). Importantly, while PHNs possess broad expertise in health promotion and prevention related to adolescent health, they do not necessarily have sport-specific health competence.

In 2022, there was a Norwegian political call for the sports community to develop and implement measures aimed at preventing EDs (The Norwegian Parliament, 2023). In response, the Norwegian Sports Federation has developed an action plan for the prevention of EDs in sports, which has been submitted to the Ministry of Culture and Equality for review (The Norwegian Sport Federation, 2022). The report highlights PHNs as key professionals working with young athletes in schools, emphasizing the need for them to possess the competence to identify and promptly refer individuals who may require professional assistance (The Norwegian Sport Federation, 2022). However, studies show that healthcare workers, including coaches, physiotherapists and PHNs, often have limited knowledge of EDs, and that few schools have specific procedures to deal with EDs (Dahlgren et al., 2023b; Gillbanks et al., 2022; Logue et al., 2020; Mancine et al., 2020; Martinsen et al., 2015). Similarly, the knowledge of LEA and REDs among physiotherapists and coaches is limited (Gillbanks et al., 2022; Logue et al., 2020), but it is currently unknown if PHNs frequently consulting adolescent athletes have knowledge of and feel competent to identify and address symptoms of REDs.

Hence, the aim of this study was to explore PHNs’ level of knowledge and perceived competence in identifying and addressing symptoms of EDs and REDs among young athletes at Norway's elite sport middle and high schools. Additionally, this study explored their preferred courses and methods of training.

## Method

### Study Design

This is an explorative, cross-sectional study arranged between November 2024 and February 2025.

### Sample

This study recruited PHNs working at all elite sports middle and high schools in Norway. Elite sports middle and high schools provide facilities for students who wish to invest in sports at a high level and, at the same time, wish to study. The Ministry of Education has commissioned the Norwegian Olympic Sports Centre to quality-assure the approval of private sports schools that apply for additional funding from the state (Olympiatoppen, 2019).

The inclusion criterion was being a PHN working with athletes at elite sports middle- and high schools in Norway, and no other specific exclusion criterion was defined. An email with a link to an informed consent form was sent out to all PHNs at Norway's 54 registered elite sports middle and high schools. This included a total of 58 PHNs identified by separate surveys of the various schools’ websites. The PHNs were contacted personally by mail, and reminders were sent for delayed responders. Additionally, sports directors or rectors for each of the national school network administrations were contacted and asked to inform their associated PHNs. If consenting to participate in the survey, the respondents were sent automatically to the questionnaire.

### Measures

Data were sampled using a digital questionnaire. The questionnaire “*Public health nurses’ competence about eating disorders and REDs in young athletes*” was the main tool in this study, based on a previously designed questionnaire to measure ED and LEA/REDs knowledge among sport coaches (Løvestam et al., 2024; Mathisen et al., 2025). The current form adaption was pilot tested by two PHNs employed at Oslo Metropolitan University. The questionnaire includes both multiple-choice and obligatory open-ended questions, allowing respondents to elaborate on their answers. The assessment includes both self-reported and expert-evaluated knowledge questions *(see supplementary file 1)*.

#### Knowledge Scores

Self-reported knowledge was measured using a 10-point Numeric Rating Scale (NRS) based on the visual analog scale (Hayes, 1921). Participants rated their agreement with statements (e.g., “I know what REDs is”), and the questionnaire consisted of four items for ED (definition, risks, consequences, symptoms) and five items for LEA/REDs (definition of LEA, definition of REDs, risks, health consequences, performance consequences), respectively. The mean score for each of these items is reported as overall LEA/REDs score, and overall ED score, respectively. Additionally, one item asked for their awareness of guidelines for addressing symptoms of ED or LEA, respectively, and was rated similarly.

Expert-evaluated knowledge is assessed through a 3-point NRS ranging from 0 (no knowledge) to 2 (high knowledge) based on the quality of responses to open-ended questions (e.g., “Please explain what REDs is in your own words”). The open-ended questions had a word limit of 200 words. The open-ended questions were assessed separately by three of the researchers following a scoring manual before this expert panel met for a calibration meeting on their scorings. Similarly to self-reported knowledge, an overall mean expert-assessed score was given for ED knowledge and LEA/REDs knowledge, respectively.

#### Attitudes Towards and Communication About Body Mass

Respondents rated 14 items by a Likert Scale ranging 0 (strongly disagree)–10 (strongly agree) about their attitudes towards the impact of body mass for performance in sport and about how they communicated about body mass and appearance. While it is difficult to establish strict criteria for determining the most appropriate response, the total score would typically reach a maximum of 80 when responses are most favorable (six items most favorably scored 0, with eight most favorably scored 10) (*see supplementary file 1*).

### Ethical Considerations

The study was submitted as part of a master thesis in June 2025 (embargoed for 1 year) by the Oslo Metropolitan University. The project has been assessed with regard to General Data Protection Regulation by SIKT (18.07.2024, ID number 600657). All respondents had to sign an informed consent form before being included in the study.

### Analysis

Results were analyzed by IBM SPSS statistics version 28.0.1.0 (142) and visually inspected and statistically evaluated for normal distribution. Students T-test or Mann–Whitney U test or Wilcoxon Signed Rank Test were used as appropriate to compare knowledge scores between groups within the sample (work experience less than 10 years or ≥10 years, or having attended courses on LEA/REDs or not). Correlation between self-reported knowledge and expert-assessed knowledge was explored by Pearson correlations or Spearman's Rho as appropriate. Due to the explorative nature of this study, analyses were evaluated at a significance level of 0.05. Average values are reported as mean (standard deviation, SD) or median (interquartile range, IQR) as appropriate, and effect sizes are given as Hedges g. The results are presented as average scores for the total sample, and per two group comparisons. Please see Table 1 for details on numbers per groups.

**Table 1.** Self-Reported and Expert-Assessed Scores for the Total Group and Separated According to Work Experience and Course Attendance, Respectively. Values are Mean (SD) and [95% CI] If Not Otherwise Stated.

		Self-reported, Score range 0–10		Expert-assessed, Score range 0–2	
		Overall LEA/REDs knowledge	Overall DE/ED knowledge	Overall LEA/REDs knowledge	Overall DE/ED knowledge
Total group (*n* *=* *22*)		4.6 (2.3) [3.6–5.6]	7.3 (1.7) [6.5–8.0]	0.9 (0.5) [0.7–1.3]	1.9 (0.5) [1.7–2.2]
Work experience	<10 years *(n* *=* *12)*)	4.5 (2.3) [3.0–6.0]	7.6 (1.8) [6.5–8.8]	1.1 (0.8)*	2.0 (0.6)*
	>10 years *(n* *=* *10))*	4.8 (2.3) [3.1–6.5]	6.8 (1.5) [5.7–7.8]	0.8 (0.6)*	2.0 (0.8)*
	Differences, Mean (SE), *p, g*	0.3 (0.9), [-1.7–2.4] *p* *=* *0.7*, *g* *=* *0.1*	0.9 (0.7), [-2.4–0.6] *p* *=* *0.2*, *g* *=* *0.5*	U = 50.0 *p* *=* *0.5*	U = 57.0 *p* = *0.8*
Previous course attendance	Course attendee	LEA course^1^	ED course^2^	LEA course^1^	ED course^2^
		6.4 (1.8) [4.5–8.2]	7.1 (1.5) [6.4–7.8]	1.3 (0.3) [1.0–1.6]	1.5 (0.3) [1.4–1.7]
	Non-course attendee	4.0 (2.1) [2.8–5.1]	5.9 (2.8) [1.4–10.4]	0.8 (0.5) [0.5–1.0]	1.4 (0.7) [0.2–2.6]
	Differences, Mean (SE), *p, g*	2.4 (1.0) [0.3–4.4] *p* *=* ***0.03*** *g* *=* *1.1*	1.2 (1.0) [-3.2–5.5] *p* *=* *0.5* *g* *=* *0.6*	0.5 (0.2) [0.1–0.9] *p* *=* ***0.02*** *g* *=* *1.2*	1.0 (0.2) [-1.0–1.3] *p* *=* *0.8* *g* *=* *0.3*

*Note: DE, disordered eating; EDs, eating disorders; LEA, low energy availability; REDs, relative energy deficiency in sport SD,* standard deviation; g, Hedges g; *, median (IQR), IQR, interquartile range*; ^1^LEA course attendance, n* *=* *6 (versus 16 non-attendance); ^2^ED course attendance, n* *=* *18 (versus 4 non-attendance).*

## Result

A total of 22 responding PHNs are included in this study, all female (100%) of mean (SD) age 47.8 years (10.0, 95% CI 43.3–52.2) from various sports middle and high schools in Norway. The response rate was 38%, of which 10 (45%) worked in private elite sport schools and 12 (55%) in public elite sport schools.

Non-responding schools did not differ from the included schools in terms of school networks (*p* *=* *0.149*), being private or public (*p* *=* *0.349*), or urban/rural location (*p* *=* *0.108*).

Among the respondents, 14 (65%) had personal experience with sport, with most having participated in team sports (n = 11, 50%) and/or endurance sports (n = 6, 27%), and one (4%) from esthetic or technical sports, respectively.

### Work Experience and Conditions

The group was evenly split between those who had worked less than 10 years (n = 12, 55%) and those who had worked more than 10 years as school nurse (n = 10, 45%). More specifically related to elite sport schools, most PHN's (n = 13, 59.1%) had 0–5 years of experience from sports middle and high schools, while 18.2% (n = 4) had 5–10 years, 13.6% (n = 3) had 10–15 years, and 4.5% (n = 1) had more than 15 years of experience.

The median (IQR) number of days the PHN had services at the elite sport middle schools with adolescents aged 12–16 was 1.0 (2.0) days per week, and correspondingly 2.0 (2.0) at the elite sport high schools with adolescents aged 16–19. Nine (41%) of the PHNs reported that they felt understaffed, and 13 (59%) of the PHN responded that they felt a need for more resources at their school.

**Figure 1.**
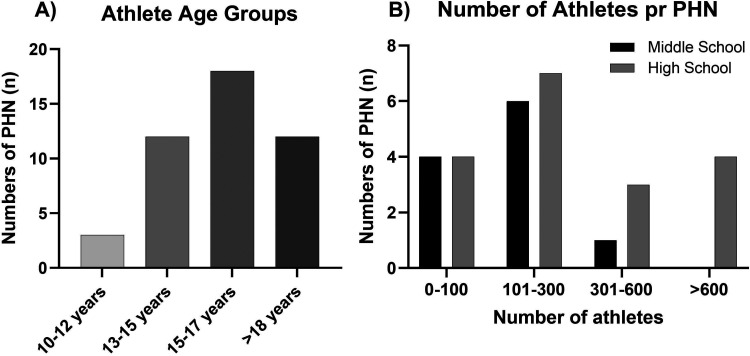
Athlete Age Groups (A) and Number of Athletes Per School Level (B) Covered by the PHNs, Reported as Number of PHN (n). PHN, public health nurse.

The age distribution of pupils that the PHNS were seeing and the number of athletes they were serving are illustrated in Figure 1.

### Courses and Education on EDs and LEA/REDs

**Figure 2.**
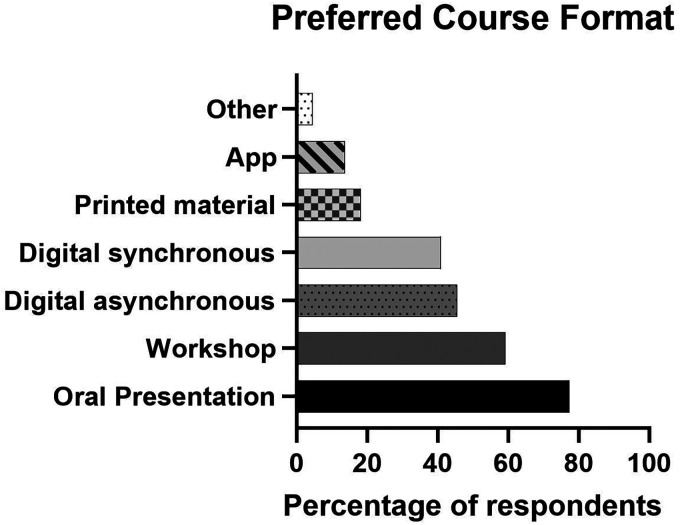
Preferred Course Format, Reported as Percentage (%) PHN. Note: digital asynchronous, self-study material like recorded lectures; digital synchronous, oral presentation in live format online; printed material, books, reports, and similar; app, self-study interactive smartphone application. PHN, public health nurse.

A total of 5 (23%) PHNs had heard about the IOC consensus report on REDs, though none had read it themselves. Six (27%) PHNs had attended courses on LEA/REDs, whereas 20 (91%) expressed an interest in attending such courses. Similarly, 18 (82%) had attended courses on EDs, whereas 20 (91%) had interest in attending courses on EDs. The preferred type of course format is illustrated in Figure 2.

### Preventive Measures Against Body Dissatisfaction by Each PHN

A total of 13 (59%) PHNs reported that they had implemented measures to prevent body dissatisfaction or enhance body acceptance, to address body image pressure, or to mitigate EDs. These measures included conversations, educational sessions, nutritional counseling, and providing information to parents and teachers.

The PHNs were asked about what strategies they used to promote a culture of openness around sensitive topic. They responded that they strive to be visible by introducing themselves in class, providing information on various health-related subjects, building trust through these interactions, and informing students about their duty of confidentiality.

### Knowledge Scores on EDs and LEA/REDs

The overall knowledge scores from self-reported knowledge (range 0–10) and the expert-assessed scores (range 0–2) for DE/ED and for LEA/REDs are presented in Table 1. There was a significant higher mean (SD) score in self-reported ED-knowledge versus self-reported LEA/REDs knowledge by 2.6 points (1.9, 95% CI 1.7–3.5), *p* < 0.001, *g* = 1.3. Similarly, higher score in expert-assessed ED-knowledge versus LEA/REDs knowledge was attained, by 0.6 points (0.6, 95% CI 0.3–0.8), *p* < 0.001, *g* = 1.0.

**Figure 3.**
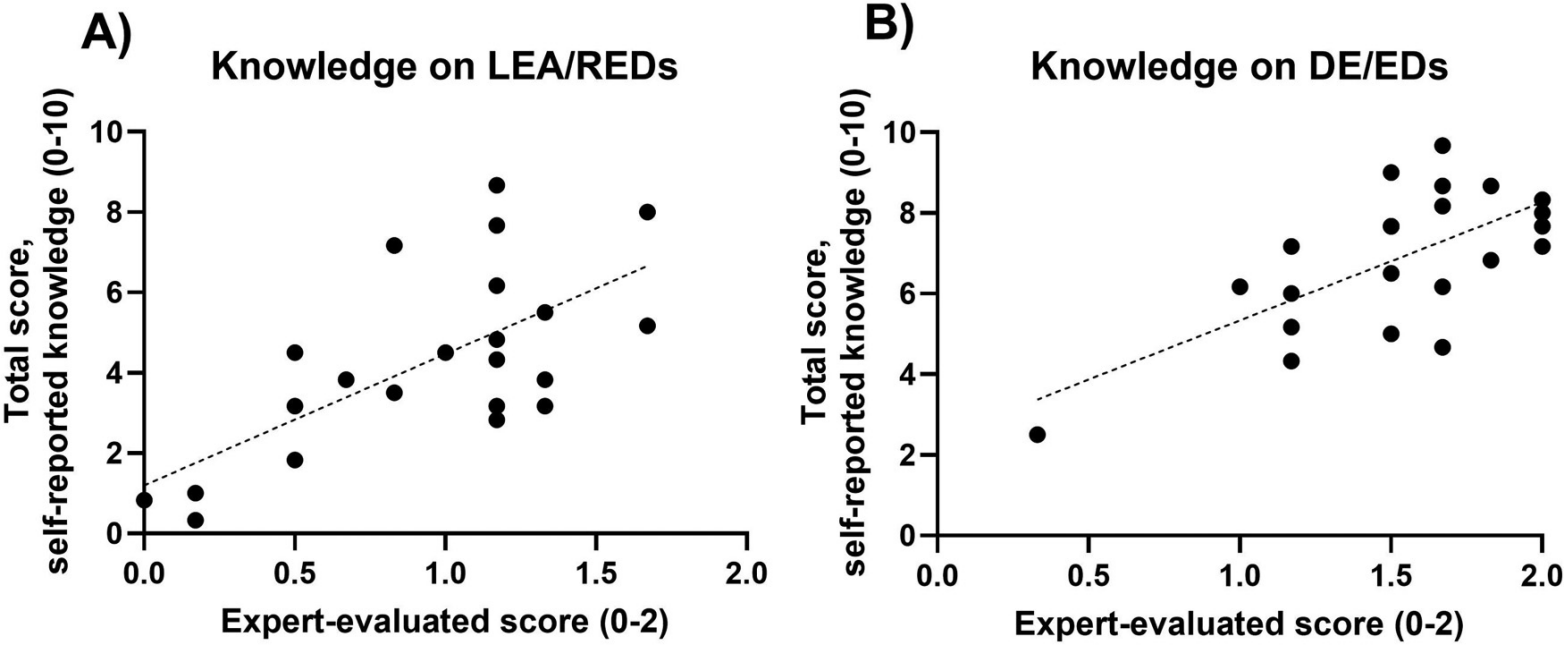
The Correlation Between Self-Reported Knowledge Score and Expert-Assessed Knowledge Score on LEA/REDs (Figure 3A) and Correspondingly for DE/EDs (Figure 3B). ED, eating disorder; RED, Relative Energy Deficiency in Sport; DE, disordered eating.

The correlation between self-reported and expert-assessed LEA/REDs knowledge was significant (r = 0.671, 95% CI 0.3–0.8, p < 0.001), indicating a moderate positive relationship (Figure 3A). Similarly, the correlation between self-reported and expert-assessed DE/ED knowledge was significant (r = 0.673, 95% CI 0.3–0.8, p < 0.001) (Figure 3B), suggesting a moderate positive relationship.

### Competence on how to Approach an Athlete with Symptoms of ED or LEA

The median (IQR) scores for self-reported knowledge on how to approach an athlete with symptoms of EDs or with LEA/REDs (range 0–10) were 5.0 (3.0) and 1.0 (4.0), respectively (*p* = 0.006). Concurrently, the corresponding expert-assessed scores (range 0–2) were 1.0 (1.0) and 1.0 (2.0) (*p* = 0.07).

The PHNs who had participated in LEA courses versus no course attendance scored significantly higher in self-reported knowledge score on how to address concerns for symptoms of LEA in athletes (U = 9.5, *p* = 0.003), whereas no difference was found for expert-assessed competence (U = 28.0, *p* = 0.1). Regarding the comparative analyses on attendance of ED courses, no significant differences were observed.

### Attitudes Towards the Importance of Body Mass in Sport

The PHNs reported a mean (SD) total score (range 0–140, ideally 80) for attitudes on BM communication of 73.5 (14.3, 95% CI 67.2–80.0), with a minimum value of 24.0 and a maximum of 99.0.

The detailed scores (range 0, strongly disagree – 10, strongly agree) were about a median (IQR) score of 5.0 (1.0) on the assumed importance of body mass for performance in sport, with an associated score of 4.0 (8.0) and 2.5 (6.0) on importance of controlling body mass in athletes within- and off-season, respectively. With respect to body composition, the median (IQR) score by PHNs on the importance of frequent assessments was 3.0 (5.0), with a corresponding score of 6.0 (4.0) for a reported assumed correlation between frequent assessments and DE/LEA. A median (IQR) score of 1.0 (3.0) represents the reported practice of providing athletes with detailed dietary information to reach a target body composition or mass. With respect to the perceived mandate to comment on an athlete's body weight in relation to the performance demands of their sport, the score was 0.0 (1.0). Concurrently, the PHNs reported a score of 9.0 (3.0) with respect to their willingness to address their concerns to an underweight athlete, and 9.0 (4.0) for addressing their concerns to athletes who frequently varies in body mass. Both the score for refraining from giving positive comments and the score for refraining from giving negative comments about an athlete's body were 10 (1.0) The PHNs reported a median (IQR) score of 5.0 (7.0) on the routines for having conversations with athletes to ensure they are doing ok.

## Discussion

This study indicated that while PHNs working at elite sport high schools demonstrate satisfactory knowledge of EDs, their understanding of LEA and REDs was insufficient. Of particular concern was their limited competence in approaching athletes with symptoms of LEA, in stark contrast to their comparatively higher competence in addressing symptoms of EDs. Work experience or participation in ED course did not influence their ED-knowledge scores, whereas participation in courses on LEA had significant positive impact on LEA-knowledge scores. Most participants reported a demand for courses on LEA, and showing a clear preference for formats that included expert-led sessions, whether delivered synchronically online or in person. In contrast, self-directed formats such as reading literature or using learning apps were less favored.

Both self-rated and expert-rated knowledge scores on EDs were high, with a strong correlation demonstrating a clustering of respondents at the upper end of the scale, with little variation. This may reflect the inclusion of highly competent health personnel who are particularly attuned to common health issues affecting their target population—adolescents. The observed difference in knowledge scores between PHNs and sports coaches (Mathisen et al., 2025) may illustrate this heightened level of competence among healthcare professionals. Conversely, while the correlation between self-rated and expert-rated knowledge on LEA/REDs was statistically significant, self-reported knowledge varied widely around the midrange of the correlation spectrum.

Furthermore, participation in courses on LEA had a significant positive effect on self-reported knowledge scores and on self-reported competence in addressing symptoms of LEA, with PHNs who had completed such courses scoring significantly higher than those who had not. Compared to sports coaches, this increase in knowledge following course participation appeared more pronounced among PHNs (Mathisen et al., 2025), potentially highlighting a greater need for sport-specific knowledge and training within the PHN population. The lack of LEA/REDs in curriculum of PHN studies is further demonstrated by a cross-sectional study of national study program for PHNs (Laholt et al., 2022).

Noteworthy, as knowledge on EDs appeared significant among PHNs, their articulated responses on knowledge showed little variation, with most identifying low body mass or weight loss as the most typical symptom of EDs. This suggests a limited understanding of EDs, which can include diagnoses without weight loss, sometimes even with significant weight gain, and often without any noticeable fluctuations (Keski-Rahkonen & Mustelin, 2016b). This is particularly relevant for individuals who are expected to gain body mass as they progress through puberty. Additionally, LEA may occur without changes in body mass and can sometimes be a consequence of EDs. Consequently, opportunities for early detection and intervention may be missed.

Based on their responses to various statements regarding attitudes toward body mass and performance, it was evident that PHNs were aware of how appearance-related comments and detailed dietary advice can contribute to body dissatisfaction and the development of EDs. In contrast, their understanding of how monitoring body mass and composition might similarly contribute to such issues—including LEA and REDs—appeared to be less robust.

The disparity in knowledge between EDs and LEA/REDs was further evidenced by respondents’ greater self-reported competence in addressing EDs, and contrasted with notably low confidence in managing symptoms of LEA. The limited knowledge of LEA/REDs was underscored by what appears to be a deflection of responsibility by the PHNs, who sporadically reported that this falls outside their professional scope and should instead be addressed by the coaching staff. This contrasts with the national guidelines for school health services by the PHNs, which underscores the importance of “*A collaboration [between school and the PHN] based on structure, clear division of responsibilities, and well-established routines with defined roles*” (The Norwegian Directorate of Health, 2017).

One way to understand these shifts in responsibility may be through how REDs are portrayed in the media —not as mental health concerns or signs of DE/EDs, but rather as the result of too much training, and thus something for coaches to manage. Nevertheless, early detection and intervention are hindered not only by a lack of competence, but also by unclear role distribution and responsibilities. The National Professional Guidelines clearly state that PHNs should detect, address, and prevent signs of psychological distress, while also considering the underlying causes (The Norwegian Directorate of Health, 2017).

Research shows that higher psychological distress predicts higher occurrence of ED-symptoms, and that prevention strategies to address ED-symptoms are required in adolescent athletes (Sundgot-Borgen et al., 2025). Although respondents mentioned various strategies to address psychological distress and EDs—such as conversations, educational sessions, nutritional counseling, and information—there was no indication of a systematic approach, nor any reports of techniques shown to effectively influence attitudes or behavior (Albarracín et al., 2024; Hagger et al., 2020; Sundgot-Borgen et al., 2024).

PHNs engage with individuals during a particularly vulnerable stage of adolescence (ages 13–18), but have limited opportunities to establish trust, given the few days allocated to each school and the large number of students under their care. These issues have previously been highlighted as barriers to effective collaboration between PHNs and schools, where responsibilities risk becoming unclear or falling into a void (Helleve et al., 2022). Furthermore, building trustful relationships takes time, and athletes in vulnerable conditions may be reluctant to seek help if the PHN feels unfamiliar or distant. In one previous study exploring how PHN may help detect and address psychological issues, continued presence and consultations were highlighted for the needed trust and alliance with the adolescent, as it may require several meetings before the adolescents open up (Moen, 2019).

Only about a quarter of the PHNs had attended any course on LEA/REDs, while the majority had participated in training on EDs. Regardless of prior course attendance, over 90% of the total sample still expressed a need for additional training on both LEA/REDs and EDs, highlighting a strong demand for further knowledge and competence in these areas. And while research-based knowledge has been communicated through linguistic translation and national reports, these written materials appear to hold little interest or practical relevance for PHNs, as is also the case for sports coaches (Mathisen et al., 2025). This is supported by a study done on how PHNs use sources of knowledge, which shows that they have limited time to find research and that they see themselves as less competent to assess research-based knowledge (Weum et al., 2017). An important takeaway from these studies is the value of “meet-the-expert” formats, such as digital webinars and in-person workshops.

## Strengths and Limitations

The strengths of this study were the invitation of a national cohort of PHN in elite sport schools and having a team of researchers who are well-versed in the field, both in understanding the PHN role and in EDs and LEA/REDs. A limitation of this study is the low response rate among PHNs. This may be attributed to their high workload, which may leave limited time for completing surveys. Nevertheless, this underscores one finding from this study: the need for increased resources and improved availability of PHNs to adequately support the health needs of athletes in elite sports schools. Additionally, some PHNs may have been hesitant to participate, possibly perceiving the questionnaire as a test of competence—despite the clarification in the information email that this was not its purpose. If low competence contributed to non-response, the study's findings regarding limited knowledge of LEA/REDs may, in fact, be understated. Another limitation of this study is the use of a non-validated questionnaire. However, the survey was pilot tested by representatives of the target population to ensure clarity. Moreover, the results remain comparable to those of other studies that have implemented similar instruments, including some that are ongoing or not yet published.

## Implication for Practice

If PHNs are to support young athletes’ mental health, there is a need for increased competence on their specific needs as athletes. As part of this knowledge and competence development, PHNs must gain a better understanding of the sport-specific performance demands and energy requirements in sports. This is essential both to understand why young athletes are particularly vulnerable to EDs and REDs, and to enable early identification of symptoms related to these health challenges. There is also a need for a clear delineation of responsibilities, where the school's coaching staff and PHNs understand and recognize each other's complementary roles. Since REDs represent a health issue rather than solely a performance concern, addressing them should be considered a core component of PHNs’ professional competence and responsibility.

The Norwegian Directorate of Health emphasizes the need for sufficient professional competence in school health services, especially regarding mental health and psychosocial support (The Norwegian Directorate of Health, 2017). PHNs play a key clinical role in identifying subtle or hidden signs of anxiety and EDs, using their health expertise to detect early warning signs that might otherwise be missed in the school setting. Through participation in interdisciplinary teams, PHNs are well-positioned to support preventive efforts—one of their core responsibilities. Their competence and presence must foster trust and continuity, enabling early intervention and providing a safe space for students to discuss sensitive issues such as body image, self-esteem, and mental health (The Norwegian Directorate of Health., 2023; ROS, 2022).

To effectively support young athletes, PHNs’ roles should extend beyond individual consultations to include shaping the broader school and training environment. They should contribute to a school culture that promotes health, inclusion, and balance by advocating for routines that ensure adequate nutrition, recovery, and psychological well-being while counteracting harmful body ideals and performance pressures.

Rather than teaching directly about nutrition or recovery, PHNs should integrate these topics into conversations with students, promoting awareness of self-care, recovery, responsible social media use, and early recognition of REDs and DE. They must remain approachable, evidence-based, and prepared to meet students who seek help independently or are referred by others. PHNs should also help establish clear pathways for identification, management, and referral of concerns. Defined responsibilities within the school health service—and prompt referral when specialized care is needed—ensure that early symptoms of REDs or DE are not overlooked. In practice, PHNs should: contribute to a school-wide approach that promotes physical and mental well-being; maintain updated competence in REDs and related conditions; and collaborate closely with teachers, coaches, and parents to ensure coherent routine.

## Conclusion

This study found that PHNs working in sports-focused middle and high schools lack comprehensive knowledge of LEA/REDs and how to appropriately address concerns in athletes exhibiting related symptoms. PHNs who had attended courses on LEA/REDs demonstrated significantly better knowledge than those without such training, while years of professional experience as a PHN showed no significant impact on knowledge levels. To better support adolescent athletes at risk of developing REDs and EDs, specialized training and targeted educational initiatives should be implemented—ideally through “meet-the-expert” formats such as webinars and workshops.

## Supplemental Material

sj-docx-1-son-10.1177_23779608251407784 - Supplemental material for Knowledge of Eating Disorders and Relative Energy Deficiency in Sports among Public Health Nurses at Elite Sport Schools Supplemental material, sj-docx-1-son-10.1177_23779608251407784 for Knowledge of Eating Disorders and Relative Energy Deficiency in Sports among Public Health Nurses at Elite Sport Schools by Siri Heradstveit, Lisa Garnweider-Holme, Brita Askeland Winje, Jorunn Sundgot-Borgen and Therese Fostervold Mathisen in SAGE Open Nursing

sj-docx-2-son-10.1177_23779608251407784 - Supplemental material for Knowledge of Eating Disorders and Relative Energy Deficiency in Sports among Public Health Nurses at Elite Sport Schools Supplemental material, sj-docx-2-son-10.1177_23779608251407784 for Knowledge of Eating Disorders and Relative Energy Deficiency in Sports among Public Health Nurses at Elite Sport Schools by Siri Heradstveit, Lisa Garnweider-Holme, Brita Askeland Winje, Jorunn Sundgot-Borgen and Therese Fostervold Mathisen in SAGE Open Nursing
