# Supplementary material for: Knowledge of Eating Disorders and Relative Energy Deficiency in Sports among Public Health Nurses at Elite Sport Schools
Source: SAGE Open Nurs. 2026 Jan 13;12:23779608251407784. doi: 10.1177/23779608251407784 (PMC12799996; doi:10.1177/23779608251407784)
Supplement: sj-docx-1-son-10.1177_23779608251407784 - Supplemental material for Knowledge of Eating Disorders and Relative Energy Deficiency in Sports among Public Health Nurses at Elite Sport Schools [file sj-docx-1-son-10.1177_23779608251407784.docx]

## School Nurses’ Competence on Eating Disorders and REDs in Young Athletes

*This is not meant to be a knowledge test but a mapping of knowledge and possible needs, so please answer as honestly as you can.
Note: It is important that no health information is disclosed in this form.*

**Name**
**Email Address**
**Year of Birth**
**Gender**

- Male
- Female
- Other

**How long have you worked as a school nurse?**

- 0–5 years
- 5–10 years
- 10–15 years
- >15 years

**Personal Experience as an Athlete**
Have you yourself been active (competed/league play/tournaments) in a sport?

- Yes
- No

*Sport*
(Only shown if “Yes” is selected above)
Which sport(s) have you competed in? (Multiple answers possible):

- Endurance sports (e.g., biathlon, cross-country skiing, running, cycling, athletics)
- Weight-class sports (e.g., boxing, martial arts, powerlifting, weightlifting)
- Aesthetic sports (e.g., gymnastics, synchronized swimming, cheerleading, rhythmic gymnastics)
- Team sports (e.g., ball sports, hockey)
- Technical sports (e.g., table tennis, shooting, sailing, athletics)
- Other

*Other, please elaborate:*
(Only shown if “Other” is selected)

**Age Groups**
I primarily advise in the age group:

- 6–9 years
- 10–12 years
- 13–15 years
- 15–17 years
- 18 years

**Experience**
How long have you worked as a school nurse at a sports school?

- 0–3 years
- 4–7 years
- 8–12 years
- 12–15 years
- >15 years

**Elite Sports Middle School**
How many students are you responsible for at elite sports middle schools?

- 0
- 0–100
- 101–300
- 301–600
- >601

**Elite Sports High School**
Same question as above, for elite sports high schools.

**Availability at Schools**
How many school nurses do you share responsibility with at your school(s)?
How many days a week are you physically present at elite sports middle schools / elite sports high schools? (Separate question for secondary school and high school)

**Have you experienced a need for more resources in your role** (e.g., more staff or more days present at school)?

- Yes
- No
  *If needed – would you like to elaborate?*

**Have you previously attended courses/training on:**
(Multiple selection possible)

- Low Energy Availability (LEA)
- Disordered eating and/or eating disorders
- Sports nutrition
- Communication skills with athletes on nutrition, ED, LEA, and/or REDs
- Relative Energy Deficiency in Sport (REDs)
- Female Athlete Triad (FAT)
- Body image, body idealization and body pressure
- Mental health in athletic youth
- Menstruation and training
- Puberty/development and its effect on athletic skills
- None of the above

**Would you be interested in courses/training on:**
(same topics as above list + “None of these topics are relevant to me” option)

*If one or more were selected above, please elaborate:*
How should such training preferably be conducted for you/others to participate? (Select multiple if desired, but be realistic):

- Self-study via books/compendiums
- In-person lectures
- In-person workshops (interaction, exercises, discussion)
- Digital asynchronous self-study (videos, links, websites)
- Digital synchronous lessons (Teams/Zoom with live instructor)
- Self-exploration through apps (interactive learning tools)
- Other
- I don’t want more courses

**IOC REDs Consensus 2023 Update**
Are you aware of the updated IOC Consensus on REDs (October 2023)?

- Yes
- No
- Don’t know

**Have you read the updated IOC REDs Consensus (2023) or its Norwegian translation (in the Norwegian Sports Medicine or the The Journal of the Norwegian Medical Association)?**

- Yes
- No
- Don’t know

**Have you read the IOC supplementary paper on body composition assessment in athletes?**

- Yes
- No
- Don’t know

**Relevant Topics and Challenges Among Your Students**
Have you observed issues related to:
(*multiple answers possible*)

- Body acceptance
- Body pressure
- Disordered eating behavior

Options:

- No
- Not at "my" school, but elsewhere
- Yes, in middle school
- Yes, in high school

**Have your schools implemented specific preventive measures for these issues?**

- Yes
- No
  *If yes – what kind of measures?*

**Have you (health staff) tried any preventive measures for the above issues?**

- Yes
- No
  *If yes – what kind of measures?*

## The DE/EDs & LEA/REDs knowledge & awareness questionnaire. Version 1.0, 2025.

*Løvestam, Camilla Kran. MSc, Norwegian School of Sport Sciences;
Solsand, Kristine MSc, Norwegian School of Sport Sciences
Sølvberg, Nina, PhD, Norwegian School of Sport Sciences
Mathisen, Therese Fostervold PhD, Østfold University College
Sundgot-Borgen, Jorunn, PhD, Norwegian School of Sport Sciences.*

**Knowledge of Low Energy Availability (LEA)**

Rate from 0 (strongly disagree) to 10 (strongly agree). Follow-up: open text fields.

- I know what LEA is.
  → Please explain what LEA is.
- I know what REDs is.
  → Please explain what REDs is.
- I know the health consequences of LEA.
  → Name four health-related consequences.
- I know the performance consequences of LEA.
  → Name three performance-related consequences.
- I know the risk factors for LEA.
  → Name three risk factors.
- I know the guidelines for handling athletes with possible LEA.
  → As a school nurse, how should you proceed if concerned?

**Knowledge of Disordered Eating and Eating Disorders**

Similar format: 0–10 scale, followed by open response.

- I know what disordered eating is.
  → Please explain.
- I know different clinical eating disorders.
  → Name at least three.
- I know the health consequences of disordered eating.
  → Name four.
- I know the risk factors for disordered eating.
  → Name four.
- I know the symptoms of eating disorders.
  → Name three observable symptoms.
- I know the guidelines for handling athletes with possible disordered eating.
  → How should you as a school nurse proceed?

**Communication**

School culture: Rate agreement with 14 statements from 0–10:

1. Weight is a key performance factor in sports

*→ Ideally scored 5-10*

1. It’s important to monitor athletes’ weight in-season

*→ Ideally scored 0*

1. It’s important to monitor athletes’ weight off-season

*→ Ideally scored 0*

1. Frequent body composition assessments are important

*→ Ideally scored 0*

1. Body comp assessments may increase LEA/disordered eating risk

*→ Ideally scored 10*

1. I provide detailed dietary advice for weight/body goals

*→ Ideally scored 0*

1. It is natural for me as a healthcare professional to comment on the athlete’s body in relation to the demands of the sport (such as weight classes and performance).

*→ Ideally scored 0*

1. If I observe that an athlete is underweight, I address it first with the athlete in question.

*→ Ideally scored 10*

1. If I observe that an athlete’s weight fluctuates frequently, I address it first with the athlete in question

*→ Ideally scored 10*

1. If I believe that an athlete should reduce their weight, I discuss it with the athlete.

*→ Ideally scored 0*

1. I regularly talk to athletes to ensure they are doing well

*→ Ideally scored 10*

1. I avoid negative comments about appearance

*→ Ideally scored 10*

1. I avoid positive comments about appearance

*→ Ideally scored 10*

1. I focus on body function rather than appearance when communicating

*→ Ideally scored 10*

**On Creating a Culture of Openness**

How do you and your school facilitate open dialogue between school nurse and student on sensitive/difficult topics?

**Comments**
Do you have any comments, explanations, or clarifications you wish to add?
